# Validation of the Skindex-17 quality of life assessment instrument for a Brazilian population^☆^^☆☆^

**DOI:** 10.1016/j.abd.2020.03.021

**Published:** 2020-11-18

**Authors:** Marilia Formentini Scotton Jorge, Ioana Bittencourt Mourão, Camila Fernandes Pollo, Ticiane Dionízio de Sousa, Silmara Meneguin, Hélio Amante Miot

**Affiliations:** Faculty of Medicine, Universidade Estadual Paulista, Botucatu, SP, Brazil

**Keywords:** Dermatology, Indicators of quality of life, Psychometrics, Quality of life

## Abstract

**Background:**

Health-related quality of life assesses how diseases affect the daily life of people; there are several generic instruments for this assessment in dermatology. Skindex was created in 1996; it is a multidimensional instrument, aiming to encompass some psychological and social aspects not yet addressed by other questionnaires. Among its versions (Skindex-29, 16, and 17), Skindex-17 had not been validated in Brazil.

**Objectives:**

To validate Skindex-17 for use in Brazilians with dermatological diseases.

**Methods:**

This was a methodological, cross-sectional, and prospective study of 217 patients attended at the Dermatology Service Outpatient Clinic, Faculdade de Medicina de Botucatu - Universidade Estadual Paulista (Botucatu, SP, Brazil), from December 2017 to September 2019. The following were evaluated: content validity, filling time, internal consistency, dimensional structure, concurrent validity (DLQI), temporal stability, and responsiveness.

**Results:**

The sample consisted of 71% women, mean age (SD) of 45 (16) years; phototypes II−IV accounted for 95% of the cases. Cronbach's alpha coefficients were 0.82 and 0.93 for the symptoms and psychosocial conditions, respectively. A high correlation was observed with the DLQI score: symptoms (rho = 0.69) and psychosocial conditions (rho = 0.75). The instrument's two-dimensional structure was confirmed through confirmatory factor analysis. Temporal stability (ICC > 0.9) and score responsiveness (p ≤ 0.02) were verified. The instrument was shown to be feasible in clinical practice due to the content validation performed by professionals and patients, as well as the low time spent completing it (< 5 min).

**Study limitations:**

Single-center study, with patients exclusively from the public healthcare system.

**Conclusions:**

Skindex-17 was shown to be a valid and consistent instrument for assessing quality of life among patients with dermatological diseases, in Brazil. Its two-dimensional structure was confirmed.

## Introduction

In the year 2000, the World Health Organization (WHO) defined quality of life as the individual's perception of their position in life in the context of the culture and value system in which they live and in relation to their goals, expectations, values, and concerns.^1^ The concepts of quality of life in the healthcare area can encompass two spheres: generic, well contemplated by the WHO definition, and health-related quality of life (HRQoL), which encompasses two fundamental parameters – subjectivity and multidimensionality.^2^ The incorporation of HRQoL study within the healthcare area is recent, and follows the evolution of the understanding of the health-disease process over the years.^2^

Skin diseases, in general, have an important impact on HRQoL, since the lesions are located mainly on visible areas, which can affect the individual's social, affective, and emotional functioning. Within dermatology, the definition of the target outcomes to be achieved with treatment is delicate, since most diseases in the specialty are not life-threatening. This highlights the importance of assessing HRQoL through appropriate instruments as a parameter for follow-up and monitoring, in addition to defining therapeutic decisions and goals.

The measurement of HRQoL is not direct; therefore, it is necessary to use instruments based on psychometric theory to enable its assessment.^3^, ^4^ Questionnaires are the most used instruments in psychometrics, and are the tools through which the impact of a disease on HRQoL can be assessed. In the meantime, the HRQoL questionnaires must obey the basic principles of psychometry: viability, reliability, sensitivity to change (responsiveness), and temporal stability.^5^, ^6^

The plurality of dermatological diseases has an impact on different dimensions of HRQoL. In 1996, Chren et al. developed the first version of the Skindex (61 items) – a multidimensional instrument (eight dimensions) for assessing HRQoL among patients with dermatological diseases. This questionnaire aimed to encompass some psychological and psychosocial aspects not yet addressed specifically by other HRQoL instruments in dermatology.^7^ The reduced version of the instrument was published in the following year – the Skindex-29, with 29 items.^8^

In 2000, a third version of the instrument was published: Skindex-16. For its development, after analyzing the use of previous versions, it was concluded that the effects of dermatoses on quality of life could be divided into three domains: skin symptoms, effects on emotions, and effect on social and physical functions.^9^

In 2006, a new, shortened questionnaire was made from Skindex-29, called Skindex-17 (SK-17), using a statistical model based on the item response theory (Rasch analysis), unlike the reduction performed to create the Skindex-16.^10^ Some modifications were implemented for better appropriateness: instead of five scores, the responses of the items now have three scores. The items were grouped into two subscales. The first addresses emotion and functioning (psychosocial), while the other addresses symptoms. Thus, an instrument with two dimensions was obtained, totaling 17 items.^10^ Of the 12 items that remained similar in Skindex-29 and Skindex-16, nine of them are also present in Skindex-17.

As SK-17 is a reduction of Skindex-29, which had already been translated into Brazilian Portuguese, there is a published translation of the items; however, this version has not yet been validated for this population.^11^ Therefore, the aim of this study was to validate the version of the SK-17 instrument, as well as to evaluate its psychometric properties among patients with dermatological diseases.

## Methods

Methodological, cross-sectional, and prospective study, carried out with patients attended at the dermatology outpatient clinic and dermatology ward linked to the Dermatology service at FMB-Unesp (Botucatu, SP, Brazil), after approval by the Institution's Research Ethics Committee (opinion No. 2.367.912).

The study included patients attended at the dermatology outpatient clinic of the Dermatology service at FMB-Unesp (Botucatu, SP, Brazil), aged ≥ 18 years; fluent in Brazilian Portuguese; and who accepted the participation by signing an informed consent.

Dr. Mary-Margareth Chren, author of the instruments, authorized its use. The authorization for use in academic research is carried out through the Mapi Research Institute – responsible for the validation of the translation of health instruments and copyright owner of the different versions of the Skindex (https://mapi-trust.org/our-resources/questionnaires-distributed-by-the-mapi-research-trust/).

Patients were interviewed during a medical consultation, after signing the informed consent form between December 2017 and September 2019. The SK-17 and DLQI questionnaires were used; for responsiveness and retest, the questionnaires were reapplied within an interval of seven to 30 days. Clinical variables (diagnosis, duration of illness, type of illness), epidemiological variables, and sociodemographic variables (age, education, sex, phototype, and family income) were also evaluated.

The content validity coefficient of the SK-17 was calculated based on the evaluation of five judges – dermatologists, psychologists, and nurses. The judges assigned a score from 1 to 5 for each item regarding the relevance of the latent trait analysis.^12^ Likewise, a group of five study participants assessed the relevance of the assessment of the construct content for each item.^12^ Considering the evaluations performed, items that reached coefficients greater than 0.7 were deemed relevant.

The internal consistency of each dimension of the instruments (SK-17 and DLQI) was assessed using Cronbach's alpha coefficient, considering 95% CI, whose lower interval should exceed 0.8 in constructs of adequate consistency.^13^ For SK-17, Cronbach's alpha coefficient analysis was also performed if each item was excluded, in order to observe whether there would be an important improvement (> 0.1) of the instrument's internal consistency. The multidimensional internal consistency for the SK-17 was estimated by Raykov's composite reliability index.^14^ The correlation between the items (inter-item) and the total score (item-total) was calculated using Spearman's rho coefficient, considering a strong correlation when rho > 0.7.^15^

Concurrent validity was assessed based on the correlation between the scores of the questionnaires and Skindex-17 and DLQI-BRA, from Spearman's correlation coefficients (rho), which should be greater than 0.7 (strong correlation).^15^

The DLQI-BRA instrument was made available by the author for free use in academic research, according to the terms of use on the website that holds its copyright (http://sites.cardiff.ac.uk/dermatology/quality-of-life/dermatology-quality-of-life-index-dlqi/dlqi-instructions-for-use-and-scoring).

The two-dimensional structure of the SK-17 was assessed by confirmatory factor analysis. The model was adjusted using the chi-squared test (χ^2^), root mean squares of approximation errors (RMSEA), comparative fit index (CFI), and standardized residual mean square root (SRMR). The goal was to meet the following adjustment criteria: (χ^2^) p-value > 0.05; RMSEA < 0.8; CFI ≥ 0.9; SMR ≤ 0.08.^16^, ^17^

The temporal stability (test-retest) of the SK-17 was evaluated in a subgroup of 16 subjects, selected for convenience (mainly those whose follow-up consultation was near or who were hospitalized) within a range of 7−30 days between the interviews, provided there was no clinical alteration of the disease. Test-retest reproducibility was analyzed using the intraclass correlation index (ICC) for complete agreement. A 95% confidence interval (95% CI) was used; results were considered satisfactory when the lower interval was > 0.7.^18^

For analysis of responsiveness, the SK-17 score was evaluated in a subgroup of 16 patients who presented with clinical alteration of their dermatosis, within an interval of 7−30 days between the interviews. The responsiveness of the scores was tested using the Wilcoxon test.

The frequency of responses for each item of the SK-17 was assessed and a frequency diagram was constructed. The ceiling and floor effects were considered when the frequency of responses was > 50% for the maximum and minimum score, respectively.

The subjects were also classified into three groups, according to the skin disease: dermatoses with more evident psychosocial impact (*e.g*., vitiligo, melasma, alopecia) predominantly symptomatic diseases (*e.g*., venous ulcer, urticaria) or symptomatic impact and psychosocial (*e.g*., psoriasis, hidradenitis suppurativa), by a qualified dermatologist.

The behavior of the SK-17 score was analyzed within subgroups such as age, sex, educational status, time of diagnosis, and type of disease, using Spearman correlation tests (age and time of disease), Jonckheere-Terpstra (educational status), Mann-Whitney (sex), and Kruskal-Wallis (type of disease).^19^

Feasibility items of the questionnaire were evaluated, such as time of execution, rate of refusal to answer, and understanding of the items.

Data were tabulated in MS Excel 2010 spreadsheets and analyzed using IBM SPSS 25 and JASP 0.11. Quantitative variables were represented as means and standard deviations or medians and quartiles (p25 − p75), when normality was not evidenced by the Shapiro-Wilk test.^19^

Findings (differences or associations) were considered significant when p-value < 0.05.

As questionnaire validation tests require five to ten subjects for each item, the sample required for SK-17 validation, according to the classic test theory, was estimated between 85 and 170 patients.^20^

## Results

Two hundred and seventeen patients were evaluated, totaling 249 responses to the questionnaires. The clinical-demographic characteristics are shown in Table 1. Patients were predominantly female, adults, of intermediary phototypes, and married. The groups of skin diseases included in the study are shown in Table 2.

**Table 1:** Clinical and demographic characteristics of the patients included in the study (n = 217).

Variable		Value
Age (years)^a^		44.8 (15.5)
Sex^b^	Female	155 (71)
	Male	62 (29)
Phototype^b^	1	3 (1)
	2	48 (22)
	3	113 (52)
	4	45 (21)
	5	7 (3)
	6	1 (1)
Marital status^b^	Single	61 (28)
	Married	119 (55)
	Separated/widowed	37 (17)
Educational status^b^	Elementary	67 (31)
	High school	57 (26)
	College or university degree	93 (43)
Family income^b^	Up to R$1,000	27 (12)
	R$1,000 to R$3,000	81 (37)
	R$3,000 to R$5,000	50 (23)
	Greater than R$5,000	59 (28)
Disease classification^b^	Symptomatic	50 (23)
	Psychosocial	86 (40)
	Both	81 (37)
Disease duration (years)^c^		6 (2−15)

a Mean (SD).

b n (%).

c Median (p25 − p75).

**Table 2:** Diagnostic groups for patients, n (%).

Diagnosis	n	%
Psoriasis	52	24
Melasma	28	12.9
Eczematous diseases	19	8.7
Acne	17	7.8
Infectious dermatoses	17	7.8
Autoimmune bullous disease	16	7.4
Alopecia	12	5.5
Connective tissue diseases	10	4.6
Chronic ulcers	9	4.1
Rosacea	8	3.7
Photodamage	7	3.2
Drug eruptions	4	1.8
Benign tumors	4	1.8
Vitiligo	4	1.8
Hidradenitis	3	1.4
Malignant tumors	2	0.9
Urticaria	2	0.9
Granuloma annulare	1	0.5
Pityriasis rosea	1	0.5
Keloid	1	0.5

The total score of the dimensions of the SK-17 instruments, as well as the total score of the DLQI, are shown in Table 3. The impact on patient’s quality of life was mostly mild to moderate when assessed by the different instruments.

**Table 3:** Scores for the SK-17 and DLQI instruments (n = 217).

Instrument	Score, median (p25–p75)
DLQI	5 (2−10)
SK-17	
SK-17 S	5 (2−7)
SK-17 P	4 (1−9)

**Figure 1:**
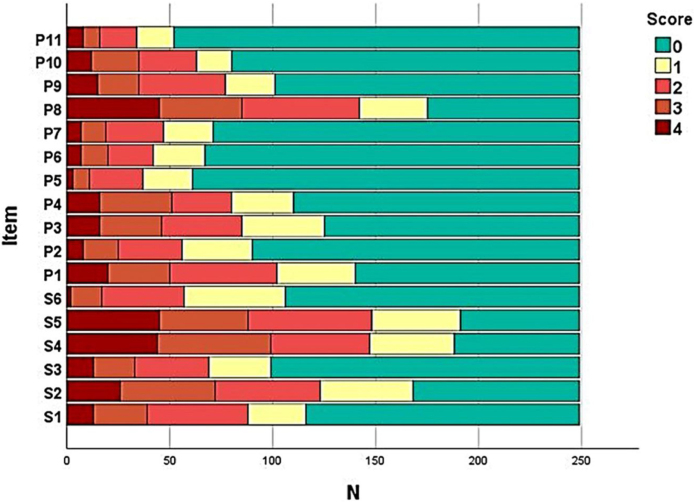
Diagram of frequency of response to items in the SK-17 (n = 217).

The frequency of each response option in each item of the instruments was evaluated (Fig. 1) in order to assess the presence of ceiling and floor effect; SK-17 presented floor effect for 13 (76%) items, especially those in the psychosocial dimension (91%).

The content of the SK-17 questionnaire was assessed by five professionals (dermatologists, nurses, and psychologist) regarding the relevance of each item for the latent trait analysis; all items presented a coefficient higher than 0.7. Cultural validation was demonstrated by the same relevance assessment with a group of five patients, resulting in mean coefficients > 0.7 (data not shown).

The analysis of internal consistency was performed for each dimension of SK-17: symptoms and psychosocial. Cronbach's alpha revealed indexes > 0.8 for the dimensions of SK-17 (Table 4). The composite reliability index (Raykov) of the SK-17 was 0.94.

**Table 4:** Cronbach's alpha for SK-17 and DLQI dimensions (n = 249).

Instrument	Cronbach's alpha coefficient (95% CI)
DLQI	0.84 (0.80−0.87)
SK-17	
SK-17 S	0.82 (0.79−0.86)
SK-17 P	0.93 (0.91−0.94)

SK-17 S, symptoms dimension; SK-17 P, psychosocial dimension; 95% CI, 95% confidence interval.

Cronbach's alpha coefficient was also evaluated for each dimension of the SK-17 in case the item was excluded, which did not demonstrate a significant variation (< 0.1) in consistency when compared with the original structure.

The inter-item and item-total correlations are shown in Table 5.

**Table 5:** Inter-item and item-total correlation coefficients (rho) for SK-17.

	S2	S3	S4	S5	S6	P2	P3	P4	P5	P6	P7	P8	P9	P10	P11	Total S	Total P
S1	0.49	0.48	0.55	0.38	0.48	−	−	−		−	−	−	−	−	−	0.75	−
S2	−	0.50	0.66	0.35	0.34	−	−	−	−	−	−	−	−	−	−	0.78	−
S3	−	−	0.51	0.32	0.36	−	−	−	−	−	−	−	−	−	−	0.71	−
S4	−	−	−	0.44	0.48	−	−	−	−	−	−	−	−	−	−	0.83	−
S5	−	−	−	−	0.34	−	−	−	−	−	−	−	−	−	−	0.63	−
S6	−	−	−	−	−		−	−	−	−	−	−	−	−	−	0.66	−
P1	−	−	−	−	−	0.65	0.58	0.65	0.51	0.57	0.50	0.55	0.62	0.51	0.47	−	0.82
P2	−	−	−	−	−	−	0.56	0.53	0.68	0.57	0.58	0.55	0.63	0.56	0.55	−	0.76
P3	−	−	−	−	−	−	−	0.54	0.49	0.51	0.45	0.60	0.61	0.59	0.38	−	0.78
P4	−	−	−	−	−	−	−	−	0.45	0.54	0.48	0.46	0.67	0.43	0.45	−	0.75
P5	−	−	−	−	−	−	−	−	−	0.60	0.60	0.47	0.55	0.52	0.52	−	0.65
P6	−	−	−	−	−	−	−	−	−	−	0.55	0.52	0.58	0.55	0.46	−	0.69
P7	−	−	−	−	−	−	−	−	−	−	−	0.48	0.55	0.53	0.53	–	0.66
P8	−	−	−	−	−	−	−	−	−	−	−	−	0.60	0.60	0.38	−	0.80
P9	−	−	−	−	−	−	−	−	−	−	−	−	−	0.61	0.50	−	0.80
P10	−	−	−	−	−	−	−	−	−	−	−	−	−	−	0.42	−	0.70
P11	−	−	−	−	−	−	−	−	−	−	−	−	−	−	−	−	0.58

S, questions of the symptoms dimension; P, questions of the psychosocial dimension; Total S, total symptom dimension score; Total P, total psychosocial dimension score.

The correlations between the instruments (rho) were strong between both the symptoms dimension of the SK-17 (0.69) and the psychosocial dimension (0.75) with the DLQI (p < 0.01).

Confirmatory factor analysis was performed to assess the dimensionality of the SK-17. Adequacy to the dimensionality proposed by the instrument was demonstrated, through agreement with all the values of the adjustment criteria (Table 6).

**Table 6:** Adjustment indexes of the confirmatory factor analysis.

Test	SK-17
Chi-square model (χ^2^)	121.81 (p = 0.14)
Mean square root of approximation errors (RMSEA)	0.02 (0.00−0.042)^a^
Comparative fit index (CFI)	> 0.99
Standardized residual mean square root (SRMR)	0.05

a 95% confidence interval.

The temporal stability of SK-17 was assessed by re-application of the questionnaires to a group of 16 patients who had no clinical variation in their dermatosis, during a period that ranged from seven to 30 days. The results obtained showed adequate test-retest reproducibility for both instruments and agreement by intraclass correlation (Table 7).

**Table 7:** Temporal stability: test-retest (n = 16); and sensitivity to change: responsiveness (n = 16) results for the study subgroups.

		SK-17 S	SK-17 P
Test-retest	1^st^ Interview^a^	3.0 (1.0−7.0)	3.3 (1.0−5)
	2^nd^ Interview^a^	3.5 (1.0−6.5)	2.5 (1.0−6.5)
	ICC (95% CI)^b^	0.98 (0.94−0.99)	0.94 (0.83−0.98)
Responsiveness	Clinic worsening^a^	7.5 (5.5−9.5)	9.0 (3.5−15.5)
	Clinical improvement^a^	5.5 (2.0−7.5)	5.0 (1.0−10.0)
	p-value	0.01	0.02

ICC, intraclass correlation coefficient; 95% CI, 95% confidence interval; SK-17 S and P, symptoms and psychosocial subscales.

a Median (p25 − p75).

b All comparisons resulted in p < 0.01.

The responsiveness of the instruments was also assessed, by reapplying the questionnaires to another subgroup of 16 patients who presented clinical dermatological changes. The analysis revealed unidirectional worsening or improvement for the dimensions of the SK-17 (Table 7).

The comparison of the scores of the two dimensions of the SK-17 with the variables studied (age, sex, education, type of disease, and time since diagnosis) are shown in Table 8. A difference was observed in scores regarding sex within the psychosocial dimension, as well as for the type of disease and for educational status within the symptoms dimension.

**Table 8:** Performance of the subgroups in the SK-17 (n = 217).

Variable	SK17-S		SK17-P	
	Parameter	p-value^a^	Parameter	p-value^a^
**Age**^b^	-0.08	0.26	-0.08	0.22
**Sex**^c^		0.25		**< 0.01**
Female	5 (2−8)		5 (2−10)	
Male	4 (2−6)		2 (0−7)	
**Educational status**^c^		**0.04**		0.33
Elementary school	5 (3−8)		4 (2−10)	
High school	5 (3−8)		5 (1−10)	
University education	4 (2−7)		4 (1−9)	
**Disease classification**^c^		**< 0.01**		0.19
Symptomatic	7 (5−9)		4 (1−8)	
Psychosocial	2 (1−5)		4 (1−8)	
Both	5 (3−8)		5 (2−12)	
**Time since diagnosis**^b^	-0.10	0.16	-0.03	0.63

SK-17 S and P, symptoms and psychosocial subscales.

a p-value, data not adjusted.

b Spearman's rho.

c Median (p25−p75).

The feasibility of the SK-17 was assessed according to the questionnaire's response time and difficulties during its completion. Response time was measured for a group of 17 patients, ranging from 58 s to 4 min (mean =1 min 48 s, SD = 42 s). No patients refused to answer the questionnaire and no items were left unanswered. No major difficulties were observed in interpreting and responding to SK-17 items, even among patients with lower educational status.

## Discussion

The psychometric assessment of the SK-17 indicates that the instrument is feasible, reliable, and valid for assessing the impact on HRQoL of Brazilian patients with skin diseases. This series included patients with dermatoses that are among the most prevalent skin diseases in Brazil, according to a recent survey conducted by the Brazilian Society of Dermatology.^21^

The impact of dermatological diseases can be assessed through instruments, divided into clinical and psychometric parameters.^2^ As many chronic dermatological diseases have as their inherent characteristic the predominant impact on HRQoL and not survival, the study and improvement of HRQoL instruments are essential for clinical practice and therapeutic trials.

The skin is the most important organ for the formation of self-image and interaction with the external environment; alterations in it can cause significant influence on daily activities.^22^ The presence of dermatoses has been shown to be related to the increased risk of suicide in some studies,^23^ and may cause greater concern in individuals than diseases such as hypertension and diabetes.^24^ The studied population was composed of young adults, a socially and professionally active age group, *i.e.*, with more presumed interpersonal interaction. The predominance of women coincides with most studies in the literature in dermatology, since women tend to seek healthcare services earlier and more often.

The assessment of HRQoL encompasses different spheres of the life of the patient, which justifies the multidimensional structure of the instruments. In this study, the two-dimensional structure of the SK-17 (symptoms and psychosocial) was confirmed, the statistical analysis demonstrated the adequacy of all adjustment indexes studied (chi-square, RMSEA, SRMR, CFI) for the dimensionality described by the authors of the original questionnaire.^9^, ^10^

The validity of the SK-17 was evidenced by its internal consistency, concurrent validity, temporal stability, responsiveness, feasibility, content validity, and cultural adequacy.

Concurrent validation performed through the correlation of SK-17 with DLQI-BRA showed adequate correlation indexes, suggesting that they evaluate the same latent trait. DLQI is the most widely used instrument for assessing HRQoL in dermatology worldwide, and was strongly correlated (> 0.7) with both dimensions of the SK-17.

The internal consistency of the SK-17 questionnaire demonstrated an adequate reliability index for its two dimensions, suggesting precision in the measurement of its scores in this population.^13^ The original SK-17 study^10^ showed Cronbach's alpha indexes > 0.7 for its two dimensions, and performed the analysis of the coefficient for each item, without improving the performance of the instrument excluding any item, which also was observed in the present work.

The temporal stability of SK-17 showed concordant scores for the same patient who presented no alteration in their dermatosis, which allows the conclusion that the instrument is reproducible in clinical practice. Likewise, the responsiveness indicators showed favorable results, reinforcing their sensitivity to change in clinical practice.

The ceiling and floor effect analysis is not always described in validation studies. A significant number of items with floor effect were observed in the SK-17. The scores of all the instruments evaluated show that the study population was composed of patients with dermatoses with mild to moderate quality of life impairment, which can contribute to null responses. As the usual HRQoL impairment in dermatology is mild and moderate, the floor effect phenomenon has also been described in other studies involving the DLQI-BRA, and may not be a characteristic of the instrument, but rather of the studied population.^25^, ^26^, ^27^

The feasibility of using the SK-17 was evidenced by the short time spent to answer the questionnaire, as well as the adequate understanding of the questions. During the Skindex-16 validation in Brazil, it was observed that the questionnaire response time was also short and adequate, ranging from two to three minutes (mean: 2 min 41 s; SD = 51 s), with a high rate of non-response to the item 5 (Persistence/recurrence); such difficulty was not observed in the present study, nor was any item left unanswered.^28^

The subgroups were analyzed according to sex, age, educational status, time since diagnosis, and type of disease. A significant difference was identified for sex within the psychosocial domain of SK-17 and type of disease and educational status within the symptom domain of SK-17.

The present study included a predominantly female population with higher levels of anxiety and concern about self-image, which could explain the difference observed.^29^ This behavior should alert to the influence of gender when describing the impact of HRQoL on dermatological diseases, especially when HRQoL scores subside therapeutic decisions, as occurs in some psoriasis treatment flowcharts.^30^

The different behavior between subgroups of types of disease in the symptom dimension can be explained by the very symptomatic nature of the diseases studied. In the original article of SK-17, ^10^ it is described that the psychosocial scale presented a similar behavior between different diseases and severities, allowing the comparison between different dermatoses, which did not occur with the symptoms scale. In the present series, the symptoms subscale showed different functioning according to the disease studied, which guides comparisons only within the same clinical context, preferably, in the same type of disease.

When analyzing the performance of the symptoms subscale regarding educational status, higher scores were associated with primary and secondary education. Such an association can be due to the fact that these patients with lower educational level may take longer to seek care (or have less access to the healthcare system), resulting in more symptomatic diseases. Studies designed specifically to assess this type of outcome are suggested.

It is worth highlighting some of the limitations of the present study: it was a single-center study, including only patients from the Brazilian Unified Health System (Sistema Único de Saúde [SUS]) and with a sample that presented some asymmetry as to the type of disease and sex. However, these limitations do not hinder the validation of the instrument for a population in Brazil.

The comparative analysis of SK-17 with other instruments for assessing QoL, such as DLQI-BRA and Skindex-16, will allow the careful assessment of the usefulness and psychometric performance of these instruments in dermatology.

## Conclusions

Skindex-17 was shown to be a valid and consistent instrument for assessing HRQoL among patients with dermatological diseases in Brazil. Its two-dimensional structure was confirmed.

## Financial support

None declared.

## Authors’ contributions

Marilia Formentini Scotton Jorge: Approval of the final version of the manuscript; design and planning of the study; drafting and editing of the manuscript; collection, analysis, and interpretation of data; critical review of the literature.

Ioana Bittencourt Mourão: Collection, analysis, and interpretation of data.

Camila Fernandes Pollo: Collection, analysis, and interpretation of data.

Ticiane Dionízio de Sousa: Collection, analysis, and interpretation of data.

Silmara Meneguin: Approval of the final version of the manuscript, critical review of the manuscript.

Hélio Amante Miot: Statistical analysis; approval of the final version of the manuscript; design and planning of the study; drafting and editing of the manuscript; effective participation in research orientation; critical review of the manuscript.

## Conflicts of interest

None declared.
