# Erratum on “Validation of the Skindex-17 quality of life assessment instrument for a Brazilian population”^☆^

**DOI:** 10.1016/j.abd.2021.04.001

**Published:** 2021-05-28

**Authors:** Marilia Formentini Scotton Jorge, Ioana Bittencourt Mourão, Camila Fernandes Pollo, Ticiane Dionízio de Sousa, Silmara Meneguin, Hélio Amante Miot

**Affiliations:** Faculty of Medicine, Universidade Estadual Paulista, Botucatu, SP, Brazil

In the article “Validation of the Skindex-17 quality of life assessment instrument for a Brazilian population”. published in An Bras Dermatol. 2021 Jan-Feb;96(1):51-58, please consider the following corrections:

For figure 1, please consider this as the correct figure:

**Figure:**
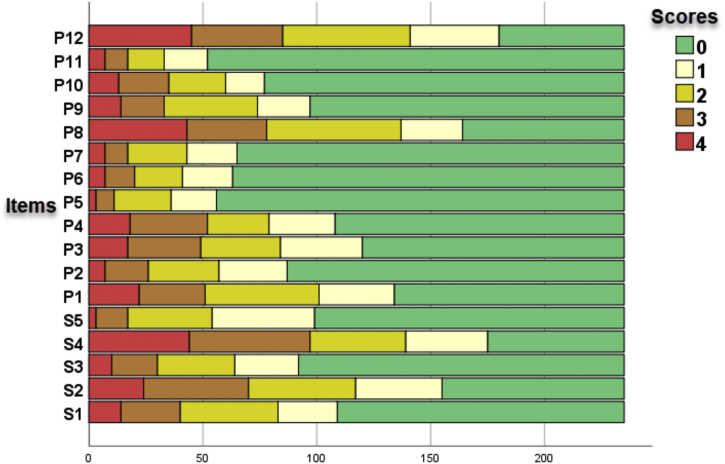

Figure 1 – Diagram of frequency of response to items in the SK-17 (n = 217).

For Table 5, please consider this as the correct table

Table 5 – Inter-item and item-total correlation coefficients (rho) for SK-17.

**Table:** 

	S2	S3	S4	S5	P2	P3	P4	P5	P6	P7	P8	P9	P10	P11	P12	Total S	Total P
S1	0.41	0.45	0.53	0.39	–	–	–		–	–	–	–	–	–	–	0.45	–
S2	–	0.43	0.64	0.32	–	–	–	–	–	–	–	–	–	–	–	0.35	–
S3	–	–	0.47	0.33	–	–	–	–	–	–	–	–	–	–	–	0.37	–
S4	–	–	–	0.42	–	–	–	–	–	–	–	–	–	–	–	0.40	–
S5	–	–	–	–	–	–	–	–	–	–	–	–	–	–	–	0.31	–
P1	–	–	–	–	0.60	0.55	0.66	0.44	0.52	0.38	0.52	0.61	0.50	0.53	0.50	–	0.78
P2	–	–	–	–	–	0.53	0.54	0.72	0.51	0.50	0.44	0.54	0.47	0.56	0.51	–	0.73
P3	–	–	–	–	–	–	0.51	0.54	0.49	0.46	0.62	0.58	0.62	0.41	0.59	–	0.77
P4	–	–	–	–	–	–	–	0.43	0.50	0.37	0.42	0.64	0.40	0.52	0.37	–	0.73
P5	–	–	–	–	–	–	–	–	0.50	0.54	0.40	0.48	0.42	0.50	0.28	–	0.64
P6	–	–	–	–	–	–	–	–	–	0.50	0.53	0.57	0.54	0.49	0.46	–	0.67
P7	–	–	–	–	–	–	–	–	–	–	0.46	0.52	0.51	0.53	0.39	–	0.61
P8	–	–	–	–	–	–	–	–	–	–	–	0.59	0.61	0.39	0.66	–	0.77
P9	–	–	–	–	–	–	–	–	–	–	–	–	0.62	0.50	0.50	–	0.78
P10	–	–	–	–	–	–	–	–	–	–	–	–	–	0.44	0.49	–	0.69
P11	–	–	–	–	–	–	–	–	–	–	–	–	–	–	0.31	–	0.60
P12	–	–	–	–	–	–	–	–	–	–	–	–	–	–	–	–	0.63

S, questions of the symptoms dimension; P, questions of the psychosocial dimension; Total S, total symptom dimension score; Total P, total psychosocial dimension score.

